# Deletion of the fungus specific protein phosphatase Z1 exaggerates the oxidative stress response in *Candida albicans*

**DOI:** 10.1186/s12864-019-6252-6

**Published:** 2019-11-19

**Authors:** Krisztina Szabó, Ágnes Jakab, Szilárd Póliska, Katalin Petrényi, Katalin Kovács, Lama Hasan Bou Issa, Tamás Emri, István Pócsi, Viktor Dombrádi

**Affiliations:** 1 Department of Medical Chemistry, Faculty of Medicine, University of Debrecen, Debrecen, Hungary; 2 Doctoral School of Molecular Medicine, University of Debrecen, Debrecen, Hungary; 3 Department of Molecular Biotechnology and Microbiology, Faculty of Science and Technology, University of Debrecen, Debrecen, Hungary; 4 Genomic Medicine and Bioinformatics Core Facility, Department of Biochemistry and Molecular biology, Faculty of Medicine, University of Debrecen, Debrecen, Hungary

**Keywords:** *Candida albicans*, Protein phosphatase Z1, Deletion mutant, Oxidative stress, *tert*-butyl hydoperoxide, Transcriptome, DNA microarray, RNA-Seq, Quantitative RT-PCR

## Abstract

**Background:**

*Candida albicans* is an opportunistic pathogen which is responsible for widespread nosocomial infections. It encompasses a fungus specific serine/threonine protein phosphatase gene, CaPPZ1 that is involved in cation transport, cell wall integrity, oxidative stress response, morphological transition, and virulence according to the phenotypes of the *cappz1* deletion mutant.

**Results:**

We demonstrated that a short-term treatment with a sublethal concentration of *tert*-butyl hydroperoxide suppressed the growth of the fungal cells without affecting their viability, both in the *cappz1* mutant and in the genetically matching QMY23 control strains. To reveal the gene expression changes behind the above observations we carried out a global transcriptome analysis. We used a pilot DNA microarray hybridization together with extensive RNA sequencing, and confirmed our results by quantitative RT-PCR. Novel functions of the CaPpz1 enzyme and oxidative stress mechanisms have been unraveled. The numbers of genes affected as well as the amplitudes of the transcript level changes indicated that the deletion of the phosphatase sensitized the response of *C. albicans* to oxidative stress conditions in important physiological functions like membrane transport, cell surface interactions, oxidation-reduction processes, translation and RNA metabolism.

**Conclusions:**

We conclude that in the wild type *C. albicans* CaPPZ1 has a protective role against oxidative damage. We suggest that the specific inhibition of this phosphatase combined with mild oxidative treatment could be a feasible approach to topical antifungal therapy.

## Background

### Medical significance of *Candida albicans*

The opportunistic pathogen *Candida* yeast colonizes the human body causing slight or undetectable symptoms in healthy individuals. However, the overgrowth of different *Candida* species causes candidiasis that may have serious consequences and poses a prominent health hazard [1]. The most common commensal yeast is *Candida albicans* [2] that is considered to be the fourth most prevalent nosocomial infectious agent in the USA [3].

The treatment of *Candida* infections has been based on the use azole drugs, first of all fluconazole [4] and echinocandins [5]. Alarmingly, around 7% of the blood samples proved to be fluconazole resistant and the echinocandin resistance was in the range of 1–2% in one study [6]. As a last resort to control severe systematic fungal infections amphotericin B can be applied, since it has a wide range of targets and generates a relatively low incidence of resistance [7]. However even this drug has its limitations, as it has toxic side effects [8]. Thus a search for novel fungal drug targets and new ways of antifungal treatments is a well justified research direction.

### A putative antifungal target

In our previous publication [8] we proposed that a specific signal transduction regulator, the *C. albicans* protein phosphatase Z1 (CaPpz1) enzyme would be a suitable drug target for the following reasons: (i) The PPZ type phosphatases are restricted to fungal species [9]. (ii) CaPpz1 has important functions as it is involved in monovalent cation homeostasis, cell wall integrity and the pathogenicity of *C. albicans* [10, 11]. (iii) The deletion of CaPPZ1 delays the yeast to hyphae morphological transition [12], and the inhibition of phosphatase could block the development of the more invasive morphological form of *Candida* [13]. (iv) The unique structural features of the CaPpz1 catalytic domain allow the design of specific inhibitors [8]. It should be added that the deletion of PPZ phosphatases in *C. albicans* [14] and *A. fumigatus* [15] made these pathogenic fungi more sensitive to oxidative stress. It is an important issue as the pathogens have to survive harsh oxidative conditions in the neutrophils and macrophages to evade the innate immune system of the host [16–18].

### The experimental approach

Based on the above grounds we decided to investigate the combined effects of the *cappz1* mutation (mimicking a specific phosphatase inhibitor) with oxidative stress (mimicking the oxidative burst inside the immune cells). After the clarification of the physiological consequences of the combined intervention we place the main emphasis on the global transcriptomic changes elicited by the phosphatase deletion and the treatment of *C. albicans* with a sub-lethal dose of the oxidizing agent *tert*-butyl hydroperoxide (*t*BOOH) alone or in combination. *t*BOOH was selected as it is a lipid-soluble organic hydroperoxide that produces peroxyl, alkoxyl and carbon-centered radicals [19]. These radicals initiate lipid peroxidation in biological membranes [20] with long-lasting cell physiological effects in fungi [21, 22]. It has been reported that *t*BOOH exposure increases the concentrations of various lipid peroxidation products including those of lipid hydroperoxides and conjugated dienes in *C. albicans* [23]. The principal technology applied in our approach was RNA sequencing (RNA-Seq) that was supplemented by DNA microarray (DNA chip) hybridization and was confirmed by monitoring the expression of a cohort of selected genes by RT-qPCR. With these three independent transcriptomic methods we could identify novel functions of the CaPPZ1 gene and reveal an interplay between oxidative stress and phosphatase deletion.

## Results

### The physiological consequences of oxidative stress and CaPPZ1 gene deletion

**Fig. 1:**
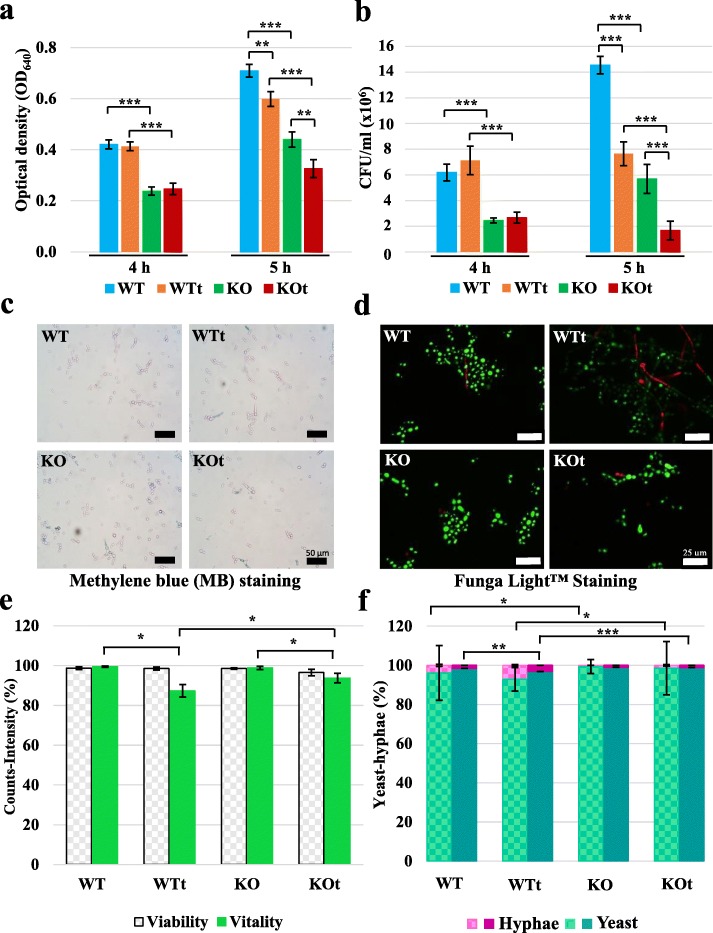
The physiological effects of CaPPZ1 gene deletion and oxidative stress in *C. albicans*. The QMY23 control and the *cappz1* deletion mutant strains were compared either under normal growth conditions (WT and KO) or after oxidative stress treatment (WTt and KOt). **a.** The effect of oxidative stress was tested by measuring the optical density at 640 nm before (4 h) and after (5 h) the additions of *t*BOOH. The mean and SD of 4 independent experiments are shown. **b.** The colony forming capacity of the same cultures was determined and evaluated as in panel a. **c.** The effect of *t*BOOH treatment on the viability of the fungal cells was analyzed by methylene blue. In the representative microscopic images, dead cells are blue and viable cells are unstained. Black bars represent 50 μm. **d.** The vitality of the *C. albicans* cells was determined by FungaLight™ staining. The representative fluorescent microscopic images shown vital cells in green and damaged cells in red. White bars represent 25 μm. **e.** Quantitative analysis of viability (checkered gray bars) and vitality (green bars) tests. The numbers of white and blue objects were counted after methylene blue staining and the intensities of the green and red objects were determined after FungaLight™ staining. The results are expressed as percentages of totals. The mean and SD of three independent experiments are depicted. **f.** The morphological forms of *C. albicans* were investigated either under the microscope (checkered bars) or by flow cytometry (clear bars). Shades of turquoise indicate yeast cells and pink show the percentage of hyphae. (Note, that only the percentage of hyphae changed significantly.) The mean and SD of 3 or 4 biological replicates are presented. The significance of the differences was estimated by two-sided, two-sample Student’s t-test and is labelled as * = *p* < 0.05, ** = *p* < 0.01, and *** = *p* < 0.001 in all panels. The experimental data used in this figure can be found in Additional file 3: Dataset S2

The characteristic phenotypes of the *cappz1* deletion mutant [10, 12] and some physiological effects of *t*BOOH treatment [14] were described earlier, but a detailed analysis of the latter has not been carried out in the QMY23 strain of *C. albicans* yet. In the present work we used the QMY23 strain for comparison (WT) since it has exactly the same genetic background as the *cappz1* (KO) [10, 24]. Based on our previous results [14] we selected 0.4 mM *t*BOOH for the stress treatment of both strains (KOt and WTt) as this concentration of the oxidative agent elicited only a moderate decline in the growth rate of the fungal cells*.* The optimal timing of the treatment was determined in preliminary experiments (Additional file 1: Figure S1). The regime of 4 h pre-culturing followed by a 1 h stress treatment was selected in order to detect the short-term response to the oxidizing conditions. The 1 h length of treatment was also comparable with the time brackets of earlier transcriptomic investigations [16, 17, 25]. The effect of this treatment on the growth rate of WT and KO strains was tested by measuring the turbidity of the samples after the 4th and 5th h of culturing (Fig. 1a). As expected, the mutant strain grew more slowly than the WT at both time points [10, 12], and the *t*BOOH treatment reduced the growth rate of both strains [14]. The colony forming capacity of the same cells was reduced by the phosphatase mutation and oxidative stress in a similar way (Fig. 1b), with the exception that after 1 h incubation *t*BOOH had a more robust effect on cell survival. The size of the growing colonies was the same in all of the samples. To test the physiological status of *C. albicans* after the treatments, we investigated the viability and the vitality of the cells. After methylene blue staining (Fig. 1c) the counting of the white and blue objects under the phase-contrast microscope proved that the oxidative stress did not affect the proportion of viable cells significantly. After FungaLight™ double-staining (Fig. 1d) the calculation of the green to red intensity ratios in the fluorescent microscopic fields (Fig. 1e) suggested that oxidative stress generated more dead, red stained cells in the WTt samples. A closer visual inspection of the images (Figs. 1c–d) hinted that at least part of changes in the staining intensities could be caused by the morphological differences between the samples. Although our cultivation conditions do not favor hyphal growth a small population of the control cells (4%) exhibited this morphology under normal growth conditions, and responded to the oxidative stimulus by elevating the proportion of hyphae (7%) in the culture (Fig. 1f). In agreement with our earlier reports [10, 12] the deletion of CaPPZ1 prevented hyphal outgrowth and there were no significant changes in KO morphology upon *t*BOOH treatment either (Fig. 1f). Consequently, the oxidative stress reduced metabolic activity in both *Candida* strains, but the higher susceptibility of the WT strain seems to be artificial due to the more intensive red staining of hyphae (Fig. 1d). From the results presented in Fig. 1 we conclude that the short oxidative stress elicited by low concentration of *t*BOOH blocked the proliferation, but did not affect the viability of the surviving cells and caused only small changes in the vitality (metabolic activity) and morphology of the treated *C. albicans* cells. Thus the samples are comparable and are suitable for subsequent biochemical analysis.

To characterize the biochemical response mechanisms of *C. albicans* to the oxidative challenge the activities of typical antioxidant enzymes and the concentrations of the oxidized and reduced forms of glutathione were determined (Table 1). The *t*BOOH treatment of the control cells elevated the activities of glutathione metabolizing enzymes and modified intracellular glutathione concentrations significantly. Interestingly, the genetic elimination of CaPpz1 had a similar (albeit somewhat smaller) effect on glutathione metabolism. In addition, the deletion doubled the activity of the antioxidant enzyme, catalase. The oxidative treatment of the KO strain resulted in the most pronounced changes in all of the measured parameters (Table 1). The positive cooperation between the phosphatase deletion and oxidative stress response was most conspicuous in the elevation of catalase and glutathione reductase activities.

**Table 1:** Comparison of antioxidant enzyme activities and glutathione concentrations of control (WT) and *cappz1* deletion mutant (KO) *C. albicans* strains after 1 h cultivation in the absence or in the presence of 0.4 mM tBOOH (WTt and KOt)

	WT	WTt	KO	KOt
Catalase [kat (kg protein)^−1^]	0.5 ± 0.07	0.5 ± 0.09	1.1 ± 0.04 ^***,a^	2.2 ± 0.15 ^***,b; ***,c^
Superoxide dismutase [munit (mg protein)^− 1^]	0.11 ± 0.02	0.11 ± 0.02	0.14 ± 0.02 ^*,a^	0.15 ± 0.02 ^*,c^
Glutathione peroxidase [mkat (kg protein)^− 1^]	0.3 ± 0.04	0.7 ± 0.2 ^**,a^	0.6 ± 0.1 ^**,a^	0.8 ± 0.1 ^*,b^
Glutathione reductase [mkat (kg protein)^− 1^]	2.5 ± 0.15	6.9 ± 1.2 ^***,a^	5.3 ± 0.8 ^***,a^	9.9 ± 0.8 ^***,b; **,c^
Reduced glutathione (GSH) [nmol (OD_640_)^− 1^]	435 ± 40	548 ± 25^**,a^	430 ± 35	525 ± 44 ^*,b^
Oxidised glutathione (GSSG) [nmol (OD_640_)^− 1^]	3.9 ± 0.4	17.9 ± 2.2 ^***,a^	8.4 ± 1.7 ^**,a^	20.8 ± 7.2 ^*,b^
GSH/ GSSG	113 ± 17	31 ± 4 ^***,a^	53 ± 13 ^**,a^	28 ± 11 ^*,b^

All data represent means ± SD calculated from four independent experiments

All of the original assay results used in this table can be found in Additional file 3: Dataset S3

^*^ *p* < 0.05, ^**^ *p* < 0.01 and ^***^ *p* < 0.001 according to Student’s t-test

^a^ Significant difference between the WTt and KO strains in comparison to the untreated WT control strain

^b^ Significant difference between the KOt and untreated KO cells

^c^ Significant difference between the WTt and KOt cells

### General evaluation of RNA-Seq results

Our findings described in the previous chapter confirm that the optimal conditions for testing the consequences of oxidative stress were correctly established. Furthermore, a pilot DNA chip hybridization experiment revealed large and reasonable alterations in mRNA levels, thus our experimental system was suitable for an extensive global transcriptome analysis. The gene expression patterns were determined in three biological replicates of the WT, KO, WTt, and KOt samples by RNA-Seq. The reproducibility of sequencing results was confirmed by clustering of the data (Additional file 1: Figure S2) and by principal component analysis (PCA, Additional file 1: Figure S3). The RNA-Seq data were compared to those of the pilot DNA chip hybridization (Additional file 1: Figure S4). All the comparisons involving oxidative stress exhibited good correlation between the two independent methods (the Pearson’s correlation coefficients were in the range of 0.65–0.71) but the KO vs WT data were practically unrelated (Pearson’s correlation coefficient: 0.04). This discrepancy may reflect the low number of the genes affected, and/or the relatively small changes of gene expression in the *cappz1* strain.

**Fig. 2:**
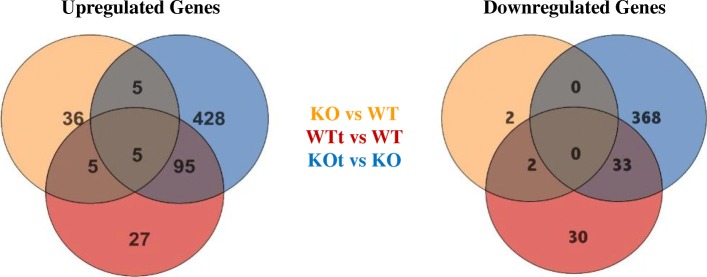
Overview of RNA-Seq data. The effects of phosphatase deletion (KO vs WT) as well as 1 h oxidative stress of the control (WT vs WTt) and on the mutant (KO vs KOt) cells are depicted in the Venn diagrams. Only the genes exhibiting more than 2-fold increase or decrease in their expression are shown. Full gene lists are available in Additional file 3: Dataset S4

All of the large (more than 2-fold) and significant transcript level changes are summarized in two Venn diagrams according to three different comparisons (Fig. 2). It is seen that the phosphatase mutation alone (KO vs WT) has only a small effect (51/4 genes up/down), oxidative stress of the control strain (WTt vs WT) affects a moderate number of the genes (132/64 genes up/down), and the oxidative treatment of the mutant (KOt vs KO) is the most effective (533/401 genes up/down). Interestingly, the numbers of upregulated genes consistently exceed the downregulated ones. Just the sheer numbers of the affected genes indicate a synergistic relationship between CaPPZ1 deletion and oxidative stress that has been forecasted by our physiological and biochemical experiments (Fig. 1 and Table 1).

### GO term enrichment analysis

**Fig. 3:**
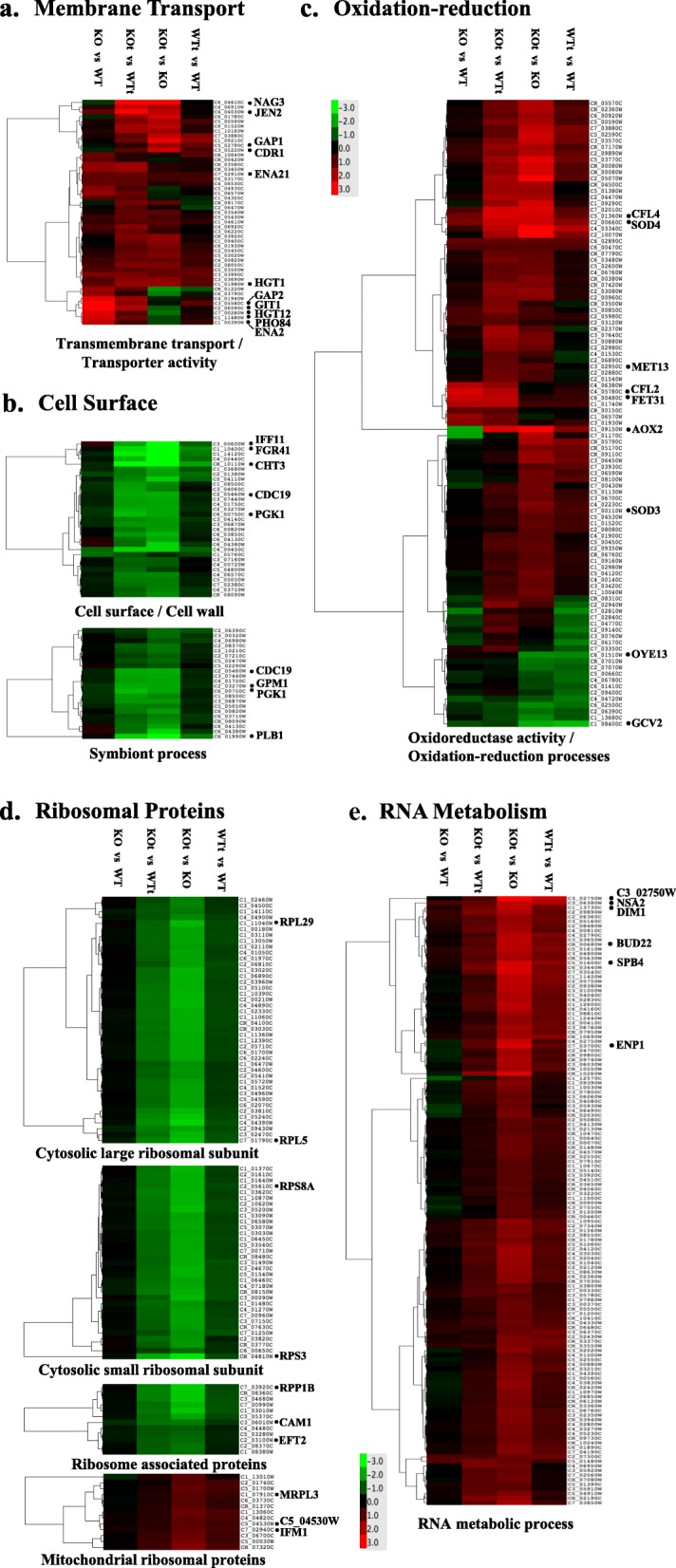
The effects of *cappz1* mutation and *t*BOOH induced stress on the gene expression of *C. albicans*. Heat maps organized in five groups (**a-e**) demonstrate the expression profiles of selected gene groups according to the color scale. Besides the comparisons described in Fig. 2, the *t*BOOH treated control cells were compared to the mutant cells treated in the same way (KOt vs WTt) in order to highlight a positive interaction between the oxidative stress and the *cappz1* mutation. RNA-Seq data from three independent biological replicates were analyzed. Black dots and systematic names indicate the genes that were selected for further analysis by RT-qPCR. The color scale indicates gene expression changes in log_2_FC units. Additional file 3: Dataset S6 summarizes the data that were used for the construction of all heat maps

Based on a full scale GO term enrichment analysis (including molecular function, biological process, and cellular component related terms) the expression changes were divided into four broad categories (Table 2). The main effect of phosphatase deletion was the upregulation of membrane transport related genes. The oxidative stress of the WT strain had a sporadic effect on cell surface, metabolism and translation associated genes. Some of these changes were enhanced and many more become significant if the stress treatment was carried out in the KO strain. A new type of comparison (KOt vs WTt) was introduced in the table to highlight a positive interaction between the effects of phosphatase deletion and oxidative stress. In order to gain a more clear-cut picture, the raw gene lists of the GO terms were compared to each other and were manually curated by eliminating false/unrelated entries, adding missing members of a family, merging overlapping terms, and dividing large groups into smaller subgroups. The revised data (Additional file 3: Dataset S6) were used to create five groups of heat maps to visualize relative expression of each gene in a subgroup (Fig. 3).

**Table 2:** Summary of GO terms enriched and the number of genes affected by the deletion of the *CaPPZ1* gene under normal (KO vs WT) or oxidative stress conditions (KOt vs WTt), as well as by oxidative stress treatment of wild type (WTt vs WT) or of phosphatase deletion mutant (KOt vs KO) *C. albicans* strains

GO ID	GO TERM	1st line: Number of up/down regulated genes			
		2nd line: Cluster frequency (%)			
		3rd line: Corrected *p* value			
		KO vs WT	KOt vs WTt	KOt vs KO	WTt vs WT
	Membrane Transport				
55085	Transmembrane transport^a^	12/0	34/0	22/3	4/1
		23.5	12.8		
		0.00614	0.01683		
5215	Transporter activity^a^	11/0	35/0	23/3	3/1
		21.6	13.2		
		0.01162	0.00212		
16021	Integral component of membrane	14/0	7/0	2/4	1/1
		7.7			
		0.00091			
	Cell Surface				
9986	Cell surface^a^	0/0	0/45	0/58	0/10
			19.1	14.5	15.4
			4.2E-21	2.38e-21	0.00297
5618	Cell wall^a^	0/0	0/38	0/50	0/9
			16.1	12.5	13.8
			2.85e-18	1.45e-19	0.00320
31012	Extracellular matrix	0/0	0/17	0/20	0/1
			7.2	5.0	
			1.95e-07	4.74e-06	
97311	Biofilm matrix	0/0	0/17	0/20	0/1
			7.2	5.0	
			1.95e-07	4.74e-06	
44403	Symbiont process^a^	0/0	0/15	0/22	0/2
				5.5	
				0.01879	
51701	Interaction with host	0/0	0/11	0/16	0/0
			4.7	4.0	
			0.01453	0.00167	
	Metabolism				
6520	Cellular amino acid metabolic process	0/1	1/0	0/4	0/11
					16.9
					0.00141
1901607	Alpha-amino acid biosynthetic process	0/0	13/0	21/0	3/0
				3.9	
				0.03642	
55086	Nucleobase-containing small molecule metabolic process	0/1	0/15	0/24	0/6
				6.0	
				0.02650	
97384	Ergosterol biosynthetic process	0/0	3/0	2/0	0/7
					10.8
					1.55e-06
6732	Coenzyme metabolic process	0/0	0/16	0/15	0/0
			6.8		
			0.04756		
6733	Oxidoreduction coenzyme metabolic process	0/0	0/14	0/15	0/0
			5.9	3.7	
			0.00026	0.04689	
16491	Oxidoreductase activity^a^	4/1	43/0	32/8	0/19
			16.2		29.2
			8.93e-06		2.42e-06
55114	Oxidation-reduction process ^a^	6/0	51/0	62/0	12/2
			19.2	11.6	
			2.04e-10	0.00131	
	Translation				
3735	Structural constituent of ribosome	0/0	0/71	0/76	0/2
			30.1	19.0	
			8.96e-60	5.71e-48	
22685	Cytosolic ribosome	0/0	0/63	0/70	0/2
			26.7	17.5	
			1.33e-65	1.51e-60	
15934	Large ribosomal subunit^a^	0/0	0/42	0/46	0/2
			17.8	11.5	
			7.71e-48	2.40e-25	
15935	Small ribosomal subunit^a^	0/0	0/22	0/24	0/0
			9.3	6.0	
			8.13e-15	7.83e-12	
30684	Preribosome	0/0	18/25	85/0	24/0
				15.9	18.2
				3.46e-45	4.31e-12
16070	RNA metabolic process^a^	0/0	27/0	132/0	34/0
				24.8	25.8
				3.36e-13	0.00792
140098	Catalytic activity, acting on RNA	0/0	7/0	41/0	8/0
				7.7	
				0.00037	

See Additional file 3: Dataset 5 for the RNA-Seq based transcriptome analysis of the individual genes

^a^Used for the construction of Fig. 3

The strong upregulation of some genes related to transmembrane transport and transporter activity either in the untreated or in *t*BOOH treated mutant strains is obvious in Fig. 3a. The genes of cell wall, cell surface, and symbiont process get downregulated in the *t*BOOH stressed KO samples (Fig. 3b). The expression patterns of the oxidation-reduction related genes are more variable (Fig. 3c), some of them are upregulated even in the untreated mutant samples, others require oxidative stress for their more intensive expression, while some are downregulated under oxidative conditions. Nearly uniform expression patterns were obtained, when the mitochondrial ribosomal protein related genes were separated from the genes coding cytosolic ribosomal subunit and associated proteins (Fig. 3d). The latter are all downregulated in the *t*BOOH treated mutant strain in a well-coordinated fashion. On the other hand, the genes of mitochondrial ribosomal subunit components and associated proteins are upregulated in the KOt samples. Interestingly, the genes associated with RNA metabolic processes and mRNA maturation follow a similar trend, their mRNAs are present at higher levels in oxidative agent treated cells, especially in the KO genetic background (Fig. 3e).

### Confirmation of RNA-Seq data by RT-qPCR

The expression of selected genes was investigated by an independent method. With the aid of the heat maps (Fig. 3) we picked typical or interesting genes from each of the main categories and added a few more genes of interest to the list of targets that were selected for RT-qPCR analysis.

From the membrane transport category (Fig. 3a) we selected 11 transporters and added TRK1 as a control. Table 3 demonstrates that in the KO strain the relative expression of the two sodium transporters ENA2 and ENA21 was upregulated together with phosphate (PHO84), glycerophosphoinositol (GIT1), and glucose (HGT1) transporter genes. The oxidative stress induced a similar elevation in the expression of HGT1 as well as the GAP1 and GAP2 amino acid transporters, and the CDR1 multidrug ABC transporter. The oxidative upregulation of GAP1, HGT1, CDR1 and multidrug NAG3 genes in the KOt samples was also substantiated. The RT-qPCR of HGT12 and JEN2 transporters was not conclusive and the mRNA level of the TRK1 potassium transporter remained practically unchanged.

**Table 3:**
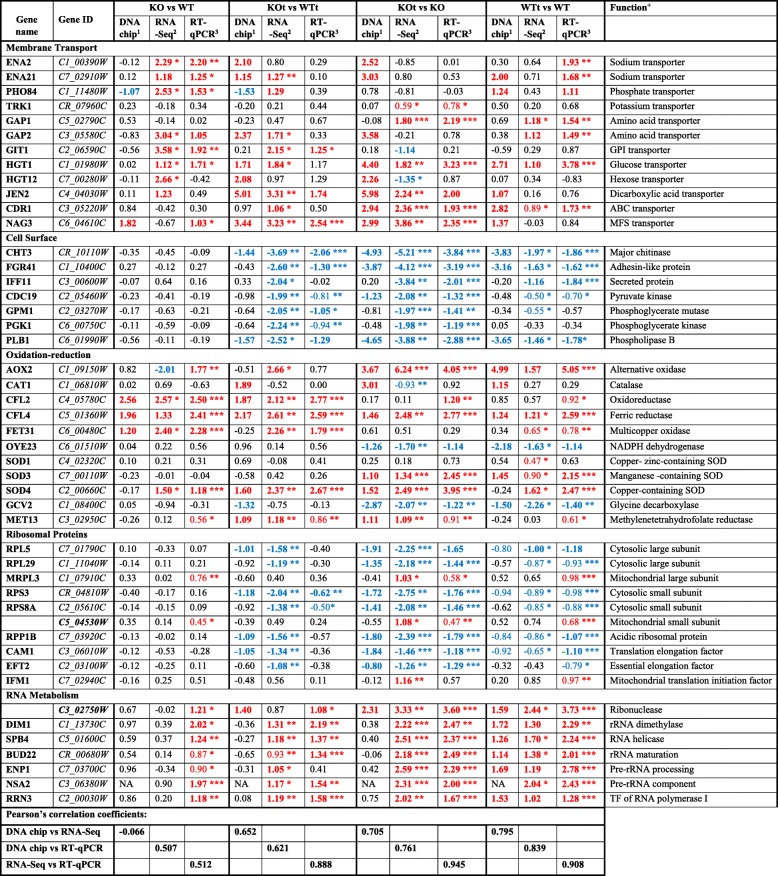
Changes of gene expression levels (in log_2_FC) in response to the deletion of the *CaPPZ1* gene under normal (KO vs WT) or oxidative stress (KOt vs WTt) conditions as well as upon tBOOH treatment of wild type (WTt vs WT) or mutant (KOt vs KO) *C. albicans* strains determined by three independent methods

More than 2-fold changes are highlighted in bold face. More than two fold and/or significant down- (blue) or upregulation (red) effects are color coded. ^*^ *p* < 0.05, ^**^ *p* < 0.01 and ^***^ *p* < 0.001 are labeled according to ANOVA test combined with Tukey post hoc test. (Note that the significance of DNA chip results cannot be determined)

NA means not applicable as the gene was not represented on the DNA chip

All of the data used for the construction of the table are presented in Additional file 3: Datasets S7 and S8

^a^Result of a single pilot experiment

^b^Mean and significance of three independent experiments

^c^Mean and significance of five independent experiments

^d^*Abbreviations*: *GPI* glycerophosphoinositol, *MSF* major facilitator superfamily, *SOD* superoxide dismutase, *TF* transcription factor

The similar expression patterns of 7 assorted cell surface related genes were confirmed by the RT-qPCR (Table 3). The proteins coded by CHT3, FGR41 and IFF11 are located at the cell surface, GPM1 and PLB1 are involved in the symbiont process, while PGK1 and CDC19 belong to both categories (Fig. 3b). The phosphatase deletion alone had no significant effect. With the exception of GPM1 and PGK1 the suppression of gene expression by oxidative treatment was detected in the wild type strain. The robust downregulation of all tested genes by tBOOH in the mutant indicated the elevated oxidative stress sensitivity of *C. albicans* in the absence of the CaPpz1 phosphatase.

From the diverse collection of metabolic genes, we concentrated on the GO term of oxidoreductase activity and/or oxidation-reduction processes that were merged in the oxidation-reduction gene group (Fig. 3c). In the group of the 9 selected representative genes GCV2, and MET13 are involved in cellular amino acid metabolism and alpha amino acid biosynthesis. Based on our biochemical tests (Table 1) two more genes, namely CAT1 and SOD1 were added to the list, so altogether 11 genes were investigated by RT-qPCR (Table 3). The mRNA levels of CAT1 and SOD1 did not change, the downregulation of GCV2 was confirmed, and the alterations in OYE23 expression were not significant. On the other hand, the mutation caused a large and significant increase in the expression of CFL2, CFL4, FET31 and SOD4 (one of the superoxide dismutase isoenzymes). The alternative oxidase AOX2, CFL4, as well as two SOD isoenzymes (SOD3 and SOD4) were upregulated in response to the oxidant especially in the KO samples.

The highly coordinated expression of cytosolic ribosomal protein genes (Fig. 3d) was confirmed by RT-qPCR (Table 3). The genes coding large subunit proteins (RPL5 and RPL29), small subunit proteins (RPS3 and RPS8A), acidic ribosomal protein (RPP1B), and translation elongation factors (CAM1 and EFT2) were all downregulated in the presence of *t*BOOH in the WT, and especially in the KO cells. In contrast, the genes of mitochondrial ribosome related genes (Fig. 3d) including large subunit protein (MRPL3), small subunit protein (C5_04530W), and mitochondrial translation initiation factor (IFM1) genes exhibited a distinct expression pattern, as they did not change or were upregulated by oxidative stress in the WT and KO strains.

RT-qPCR data support the view that the relative expression of RNA metabolism related genes (Fig. 3) is elevated by oxidative stress (Table 3). The oxidant induced upregulation was even more efficient in the phosphatase mutant than in the control strain in all of the tested cases including two genes of general RNA modifying enzymes (C3_02750W ribonuclease and SPB4 helicase), as well as the genes associated with rRNA synthesis (RRN3), maturation (BUD22, NSA2), or processing (DIM1, ENP1).

## Discussion

### Novel functions of the CaPpz1 phosphatase

The slow growing phenotype and the suppression of filamentous growth are well known consequences of the deletion of the CaPPZ1 gene [10, 12]. RNA-Seq revealed that the deletion affected only a small change in mRNA expression. Altogether 55 genes changed more than 2-fold, out of which 51 were upregulated in the KO samples (Fig. 2a). According to GO term enrichment analysis (Table 2) a large proportion of these changes were related to transmembrane transport (12 genes) and oxidation-reduction processes (6 genes).

#### Membrane transport

The increased transcription of the ENA2 and ENA21 Na-transporters (Table 3) was expected since the *cappz1* deletion mutant is tolerant against the monovalent Na^+^ and Li^+^ cations [10]. The sodium tolerance of the corresponding *ppz1 S. cerevisiae* mutant was explained, at least in part, by the enhanced Na-efflux mediated via the overexpression of the ENA1 ATP-ase [9], that is similar to the ENA2 and ENA21 P-type ATPase sodium pump genes in *C. albicans*. Thus it is likely that both ENA2 and ENA21 are controlled by the CaPpz1 enzyme. According to earlier microarray hybridization experiments [25] the incubation of SC5314 *C. albicans* cells in 0.3 M NaCl for 1 h induced the expression of both ENA2 and ENA21 genes, suggesting that the CaPpz1 phosphatase interacts with the sodium transport mediated main osmotic stress response mechanism. In analogy with *S. cerevisiae* CaPpz1 can regulate osmotic stability and cell wall integrity through an alternative pathway via the modulation of potassium transport [26]. *C. albicans* has a single ortholog of the budding yeast TRK1 and TRK2 potassium transporters, the TRK1 gene, whose expression does not change in the mutant (Table 3). Still it is possible that the phosphatase may regulate potassium influx by posttranslational protein modifications just as in the budding yeast counterparts [27].

The intriguing possibility that the CaPpz1 phosphatase may be involved in controlling the nutrient supply of the cells via the modulation of trafficking phosphate (PHO84), glycerophosphoinositol (GIT1) or glucose (HGT1) through the plasma membrane has not been explored yet. The transcriptional regulation of PHO84 by CaPpz1 is especially interesting, since a recent paper [28] states that the Pho84 protein is not only a high affinity phosphate transporter, but also a potent regulator of the Target of Rapamycin complex 1 (TORC1) pathway that coordinates cell growth with phosphate availability and other environmental clues.

#### Oxidation- reduction

The involvement of CaPpz1 in oxidative stress response was reported earlier [14], but the underlying mechanisms remained unknown. The effect of the phosphatase deletion on the antioxidant enzymes and on the glutathione (GSH and GSSG) concentrations of *C. albicans* was scrutinized in obligatory control experiments and revealed some unsuspected changes (Table 1). The KO strain exhibited a significantly elevated specific activity of all tested enzymes (including SOD) and a significantly reduced GSH/GSSG ratio. It means that CaPpz1 can modulate the oxidative status of the pathogen via the alteration of critical oxidoreductase enzyme activities. This novel finding inspired further investigations.

The GO term analysis of the RNA-Seq data indicated some gene expression changes related to oxidoreductase activity and oxidation-reduction process (Table 2), but the enrichment was not significant. After combining the two GO terms and retaining only genuine oxidoreductases from the heat map (Fig. 3c) we selected 4 genes, whose significant overexpression in the KO strain was confirmed by RT-qPCR (Table 3). Thus we proved that the synthesis/degradation of mRNA for CFL2 oxidoreductase, CFL4 ferric reductase, FET31 multicopper oxidase, and SOD4 Cu-containing superoxide dismutase enzymes can be controlled by CaPpz1. We suggest that these changes may be associated with the modulation of oxidative stress response by the CaPpz1 phosphatase. The elevation of SOD4 mRNA level can be considered as a defensive mechanism protecting the mutant cells from the more oxidizing intracellular milieu reflected by the reduced GSH/GSSG ratio. On the other hand, the upregulation of CFL2, CFL4, and FET31 oxidases points to the possible role of CaPpz1 in iron transport, since all of these 3 gene products have a ferric reductase activity that is required for the reduction of Fe^3+^ to Fe^2+^ prior to the uptake of the essential metal from the environment. In agreement with our results, the growth defect of the *cafet31* deletion mutant in low iron medium [29], as well as the upregulation of both CFL2 and CFL4 genes in low iron conditions [30] have been reported in the literature.

### Oxidative stress response mechanisms

The physiological effects of *t*BOOH treatment were originally determined with the SN87 *C. albicans* strain [14]. Here we confirmed the diminished growth and proliferation rates of the treated cells and extended earlier observations with the genetically more relevant QMY23 control strain (Fig. 1). For the present study a sublethal concentration of 0.4 mM *t*BOOH was deliberately chosen that did not influence viability and had only a small effect on the vitality and morphology of the fungus.

The transcriptomic effects of *t*BOOH stress were moderate, the mRNA levels of 196 genes were altered (132/64 genes up/down), by a factor of two or more. We would like to mention here that a 30 min treatment of the parental SC5314 *C. albicans* strain with 0.4 mM H_2_O_2_ affected 390 genes [25], the main difference stems from the fact that more genes were downregulated by H_2_O_2_ (139/251 up/down). Our GO term enrichment analysis revealed that the genes related to cell surface and cell wall, as well as metabolic processes including oxidoreductase activity were downregulated, while RNA metabolic processes, first of all in the preribosome compartment, were upregulated by the oxidizing treatment (Table 2).

#### Cell surface remodeling

The heat map of relative gene expressions (Fig. 3b) demonstrates a nearly uniform downregulation of cell wall and/or cell surface related genes in response to oxidative shock. This is a common oxidative stress response strategy of *C. albicans* that has already been observed after H_2_O_2_ treatment [18]. More importantly, we found that this basal response is greatly exaggerated when the KO cells were stressed; the number of the genes affected increased at least 5-fold (Table 2). The stress sensitizing effect of phosphatase deletion brought up additional overlapping cell surface linked GO terms, like extracellular and biofilm matrix as well as virulence related GO terms, like symbiont process and interaction with host (Table 2). All of the latter genes were united under the umbrella of symbiont process and were characterized in a separate heat map (Fig. 3c). From the similar expression patterns we concluded that the members of the two partially overlapping groups follow a common stress response and selected 7 representative members for RT-qPCR (Table 3). The quantitative evaluation of the data suggests that we have to consider two subcategories: (i) Chitinase (CHT3), the adhesion-like protein (FGR41), the secreted protein (IFF11), and phospholipase B (PLB1) are bona fide cell surface proteins that exhibit a nearly uniform, large drop in their expression upon oxidative stress. Three of these (CHT3, IFF11, and PLB1) are secreted proteins, and all of them have a fundamental role in the interaction with host that may determine the virulence of the pathogen [31]. (ii) Pyruvate kinase (CDC19), phosphoglycerate mutase (GPM1), and phosphoglycerate kinase (PGK1) are cytosolic metabolic enzymes that are also present at the cell surface, where they have a so called “moonlighting” symbiotic role [32, 33]. These genes show a smaller downregulation in WTt that becomes large and significant in KOt samples (Table 3). The interpretation of the latter results is somewhat problematic, since oxidative stress can influence either cellular metabolism [17] or the symbiont process, or both. Nevertheless, the suppression of the expression of proven or putative virulence factors by the oxidative stress, especially in the phosphatase mutant is an important finding that can be exploited in a novel antifungal therapy (see Conclusions).

#### Changes in oxidation-reduction processes

With the exception of the GCV2 glycine decarboxylase most of the interrogated genes were upregulated by the oxidative stress (Table 3). AOX2 is the inducible alternative oxidase gene of *C. albicans*, whose expression was reported to be upregulated by the oxidizing agents H_2_O_2_ and menadione [34]. In agreement with expectations this gene exhibited an exceptionally strong induction in the presence of *t*BOOH underlying the prominent role for Aox2 enzyme in the stress response mechanism. Three iron transport related oxidoreductases (CFL2, CFL4 and FET31), two superoxide dismutase genes (SOD3 and SOD4) as well as MET13 methylenetetrahydrofolate reductase were upregulated to a lesser, but still significant extent. The expression of CFL2, CFL4, FET31 and SOD4 was also induced by the deletion of CaPPZ1 gene. In addition, a positive interaction was revealed between the effects of phosphatase mutation and oxidative stress in the regulation of CFL2, CFL4, FET31, SOD4 and MET13 transcription (Table 3). From these results a convergent mechanism of action by the two perturbations at the level of oxidoreductases can be deduced.

#### Upregulation of RNA metabolism

The oxidative stress significantly upregulated 34 genes of the broad GO term “RNA metabolic processes” (that overlaps with preribosome, and catalytic activity acting on RNA terms) and the number of affected genes raised to 132, when the mutant strain was subjected to oxidative treatment (Table 2). Unquestionably, this is the largest group of genes emerging from our transcriptomic analysis (Fig. 3e). We selected 6 members from the most intensively responding cluster: 3 RNA modifying enzymes (C3_02750W, ribonuclease; DIM1, rRNA demethylase; and SPB4, RNA helicase) as well as 3 preribosome components (BUD22 for rRNA maturation, ENP1 for pre-rRNA processing, and NSA2 as a pre-rRNA complex component) and extended the group with RRN3 that acts as a transcription factor in the recruitment of RNA polymerase I to rDNA (Table 3). RT-qPCR confirmed the large and significant induction of these genes by *t*BOOH. Since three independent methods used in our study provided comparable results (Table 3), there is a strong evidence for the acceleration of RNA modifications and especially rRNA processing in oxidative stress. It is reasonable to assume that under the general repression of ribosome biogenesis, facilitated rRNA processing may help the formation of a few new ribosomes, which are needed to synthesize stress response proteins. To the best of our knowledge, this mechanism represents a novel stress response, because rRNA processing genes were typically downregulated under cell wall integrity stress [35] and cell membrane integrity stress [36].

#### Connection with membrane transport

Not expected from the GO term enrichment analysis (Table 2), but hinted by a heat map (Fig. 3a) and clearly supported by control RT-qPCR experiments (Table 3), oxidative stress induced the expression of several transporters. The GAP1 and GAP2 amino acid transporters as well as the CDR1 ABC transporter genes were significantly upregulated by the oxidative stimulus alone. In addition to these, the expression of NAG3 major facilitator superfamily transporter and MET13 reductase were also elevated by *t*BOOH in the phosphatase deletion mutant. These results indicate that oxidative stress can modulate transmembrane transport and the induction of two nonspecific transporters can be considered as an additional protective mechanism that aids the survival of the pathogen in the presence of the oxidizing agent.

### Interaction between CaPpz1 and oxidative stress response

The starting hypothesis of our transcriptomic studies was a possible positive interaction between the elimination of CaPpz1 and the oxidative stress caused by *t*BOOH that was initiated by our published data [14] and was confirmed as well as extended by our current physiological and biochemical results. The effects of combined treatment (KOt samples) were consistently greater than the effects of the two conditions alone (KO or WTt) either in the growth rate or in the cell proliferation tests (Fig. 1 a-b). The fungistatic effect of the oxidative treatment of the phosphatase deficient strain is remarkable. The same combination proved to be the most effective means of elevating the specific activity of antioxidant enzymes and reducing GSH/GSSG ratio (Table 1). Our results suggest that the redox status of the cells is unbalanced either by the phosphatase deletion or by *t*BOOH treatment, and that these two conditions act in positive cooperation with each other.

The above tendencies are clearly reflected in the changes of the transcriptome. The mutation alone effected only 0.9%, the oxidative treatment of the WT cell 3%, and the same treatment of the KO samples 15% of the 6198 ORFs in the haploid genome, respectively. The GO term enrichment analysis supports the above results; the largest numbers of the GO terms and the largest numbers of the effected genes were found in the KO vs KOt comparisons (Table 2). Numerous heat maps constructed from the curated data of the GO term analysis confirmed this conclusion in a more demonstrative graphical way on a gene to gene basis (Fig. 3). The KOt vs WTt comparison in Table 2 and in Fig. 3 tells that the oxidative stress induced more numerous and larger changes in the KO than in the WT genetic background. The most convincing evidence for a synergistic interaction between the two conditions is provided by the transcriptome changes of genes that become large and significant only in the *t*BOOH treated mutant samples. Many of these alterations have already been described in the previous chapters, but the best example concerning the ribosomal protein coding genes is discussed here.

#### Suppression of ribosomal proteins

Our RNA-Seq results suggest that protein synthesis can be modulated by oxidative stress in two different ways in *C. albicans* (Table 2): the oxidizing agent upregulates the genes of RNA metabolism (see above), and downregulates the genes of cytosolic ribosomal proteins. The latter becomes obvious only in the mutant genetic background, when 70 components of the cytosolic ribosome, i.e. practically all of the ribosomal proteins, got downregulated. After a careful manual curation of the GO term based gene lists we identified 42 large and 33 small subunit proteins of the cytosolic ribosome, and found 12 ribosome associated proteins that follow the same trend (Fig. 3e). The RNA-Seq results were confirmed by the RT-qPCR of typical examples selected from the main categories (Table 3). The proteins of the cytosolic ribosomal subunits (RPL5, RPL29, RPS3 and RPS8A), as well as the ribosome associated acidic protein (RPP1B) and the translation elongation factors (CAM1 and EFT2) show a uniform expression pattern. The sensitive RT-qPCR method revealed that all of them were repressed by *t*BOOH (Table 3). These changes were missed in the initial evaluation of RNA-Seq because the effects were small, but the downregulation become easily recognizable in the mutant genetic background as it sensitized the fungal cells to the oxidative stress. Our preliminary DNA chip hybridization data are also in reasonable correlation with the results of two other methods (Table 3). These transcriptomic results suggest that the reduced colony forming capacity of the KOt samples (Fig. 1b) can be attributed to the suspension of protein synthesis in the mutant cells under oxidative stress. Our observations are also in agreement with an earlier DNA chip based publication [17] reporting that during the early response period of macrophage engulfment a full set of translation associated genes were downregulated. The coincidence between our results vs the published data of [17] is remarkable: by RNA-Seq we detected the reduction of mRNA levels for 75 vs 54 ribosomal subunit proteins, 11 vs 23 translation initiation factors, 10 vs 11 elongation factors, and 10 vs 20 tRNA synthases in the *t*BOOH treated KO samples (not documented). The comparison of the two sets of data suggests that the shutting down of *Candida* translation during phagocytosis can be attributed to the oxidative burst inside the macrophages.

However, we noted that not all of the ribosomal proteins behave alike, 13 mitochondrial ribosomal subunit proteins and associated proteins had to be separated from the rest of the genes in order to get a clear-cut picture (Fig. 3e). The characteristic expression profiles of mitochondrial ribosomal proteins (MRPL3 and C5_04530W) and the mitochondrial translation initiation factor (IFM1) are easily distinguishable from their cytosolic counterparts (Table 3). The differential transcriptional regulation of the two ribosome types has already been reported [17] and was explained on the grounds of the essential functions of mitochondrial ribosomes during the stress conditions of phagocytosis.

### rRNA stability during oxidative stress

The contrasting expression profiles of cytosolic ribosomal protein vs RNA metabolism genes (Fig. 3d vs Fig. 3e) prompted us to investigate the possible changes in the amounts of different rRNA species during the 1 h *t*BOOH treatment. Based on a published work [37] we designed an RT-qPCR strategy for the determination of primary transcripts of the rDNA cluster as well as the intermediate and mature forms of the rRNA (Additional file 1: Figure S5). With the aid of specific primer pairs we carried out RT-qPCRs on WT, WTt, KO, and KOt samples and did not find any convincing evidence for considerable changes in the levels of the rRNA components of cytosolic or mitochondrial ribosomal subunits (Additional file 1: Figure S6) in our experimental system.

The downregulation of ribosomal protein genes and regulatory proteins was interpreted as the proof of suppressed translation in the phagocytosed *C. albicans* [17], however the lower mRNA levels do not necessarily mean a proportional elimination of ribosomes. It is known that genuine ribosomal proteins that associate with rRNA tightly are stable, while the proteins which bind to rRNA transiently are quickly degraded [38]. Our results suggest that the accelerated synthesis and processing of rRNA together with the higher stability of ribosomal proteins may ensure a relatively stable ribosome number, at least for 1 h, despite the declining mRNA levels and reduced rate of synthesis of the ribosomal proteins under the oxidative stress. Thus it is possible that even though the translation is suppressed, the ribosomal machinery remains intact during the initial stage of stress, and stays ready for a later recovery period.

## Conclusions

### Transcriptional shift in the phosphatase mutant strain exaggerates oxidative stress response

The deletion of the CaPPZ1 gene generates small changes in RNA synthesis that results in moderate phenotypical changes of slow growth and delayed morphogenesis [12]. The physiological consequences of the induction of several transmembrane transporter genes including the known ENA2 and ENA21 sodium pumps and others described in the present study remain undetected until osmotic stress conditions are applied that reveal the salt tolerance of the mutant [10]. Likewise, the shift in the GSH/GSSG mediated oxidative status and the upregulation of oxidoreductases described here will manifest in a detectable phenotype upon oxidative stress treatment [14]. Our results confirm that in the wild type *C. albicans* one of the functions of the CaPPZ1 gene product is to protect the fungal cells from oxidative damage. The absence of the CaPpz1 enzyme alone is just a mild perturbation that can be compensated by a handful of protective/adaptive transcriptional adjustments, but these moderate modifications are still important as they make the commensal fungus susceptible to oxidative intervention. One of the underling mechanism of this sensitization may relay on the partially overlapping mRNA expression changes elicited by the phosphatase deletion and *t*BOOH treatment. Here we demonstrated that the deletion mutant strain responded with a much more robust translational reprogramming to the administration of relative low *t*BOOH than the genetically matching control strain. Although the genetic and pharmacological perturbations used in our studies certainly have different mechanisms, some of their signals converge on certain targets affecting membrane transport, oxidation-reduction processes and translation. We suggest that the positive interaction between the phosphatase and the oxidative stress response could be utilized in the development of a novel antifungal strategy.

### Towards a feasible combination therapy

The effects of combined perturbation i.e. the oxidative treatment of KO samples were consistently greater than the effects of the two conditions alone (KO or WTt) either in the growth rate or in the cell survival tests. The fungistatic effect of *t*BOOH in the mutant strain was especially robust. The upregulation of protective transporters and antioxidant enzymes was accompanied by the shedding of cell surface molecules and by a highly coordinated downregulation of the cytosolic translation machinery. It is likely that the latter was the most important factor that blocked the proliferation of the opportunistic pathogen. In addition, the phosphatase deletion prevented hyphal outgrowth that was induced by the oxidative stress, but did not affect the viability of the fungal cells. These are the desired effect that would make a combination of a putative phosphatase inhibitor with a mild oxidative stressor a promising antifungal treatment. Such a treatment could keep *Candida* at bay, but would not eradicate it completely, thus would prevent an undesired repopulation of the microbiome by potentially more harmful bacterial or fungal species. Based on our structural studies the designing of a CaPpz1 specific inhibitor seems to be a realistic goal [8]. The Achilles heel of the proposed treatment is the possible side effect of the oxidative stressor. However, if it would be used in low concentration locally its combination with a specific phosphatase inhibitor might be a feasible possibility for topical applications.

## Methods

### Fungal strains and growth conditions

Two *Candida albicans* strains were used in this study: the *ppz1* deletion mutant (KO) strain [10] with the genotype *ura3Δ-iro1Δ::imm*^*434*^*/URA3-IRO1, his1Δ /his1Δ, leu2Δ /leu2Δ, ppz1Δ:: C. dubliniensis HIS1/ppz1Δ:: C. maltosa LEU2* and the genetically matching QMY23 control (WT) strain [24] with the genotype *his1Δ/his1Δ, leu2Δ::C. dubliniensis HIS1 /leu2Δ::C. maltosa LEU2, URA3/ura3Δ::imm434, IRO1/iro1Δ::imm434*. The genotypes of samples were routinely tested by colony PCR before each experiment as described previously [39] with the primer pairs of [10]. The correct genotypes of the strains used for RNA-Seq were corroborated by the lack of PPZ1 transcript in the mutant (KO and KOt) and its presence in the control (WT and WTt) samples. The expression of PPZ1 was not affected by the oxidative treatment (not documented). The culturing of *C. albicans* was carried out in YPD medium at 37 °C as reported [40], with the exception that after dilution of the pre-cultures to 0.1 OD_640_ the cells were cultivated for 4 h with shaking at 140 rpm. At this point the OD_640_ of cultures was determined, *t*BOOH (Sigma) was added in the final concentration of 0.4 mM to half of the samples and the cultivation was continued for the desired period of time. Two parallel cultures of the treated (WTt, KOt) and non-treated (WT, KO) cells were made in each independent experiment; one of which was selected for analysis after quality testing.

### Growth rate and colony forming capacity

During the culturing of the *C. albicans* cells the optical density at 640 nm (OD_640_) was determined in each hour [40] after the addition of *t*BOOH. The duration of 1 h oxidative stress was established as an optimal time period of the treatment (Additional file 1: Figure S1). The colony forming capacity of the treated cells was determined in four independent biological experiments. In the 4th and 5th hours of culturing (after 1 h treatment) 100 μl cultures were taken from each sample type. Ten-fold dilution series were made with PBS. 120 μl of 10^− 3^, 10^− 4^, and 10^− 5^ dilutions were spread on YPD agar plates in three technical replicates. After a one day long incubation at 37 °C, colony numbers were counted manually and the results were given in CFU/ml (colony number x dilution)/volume of sample in ml). The size of the colonies was determined by the ImageJ (https://imagej.nih.gov/ij/index.html) analysis of photographic images.

### Viability

Viability of the yeast cells were checked by methylene blue staining [41]. For collecting the cells 1 ml of cultures was centrifuged at 10000 g, 4 °C for 3 min. Cells were resuspended in 300 μl phosphate-buffered saline (PBS). 100 μl methylene blue (Reanal) stain (0.001 g/ml), dissolved in 2% tri-sodium citrate dehydrate (Sigma) solution was added to 100 μl of cell suspension and was incubated at room temperature for 5 min. Viability was investigated under a phase-contrast microscope (Euromex Holland) with MicroQ-W PRO camera from 200 to 500 cells per biological sample. White and blue objects were counted manually after the adjustment with ImageJ (https://imagej.nih.gov/ij/index.html) in five randomly selected fields of view in three independent experiments. Original pictures were converted into 16-bit greyscale images, the threshold value was set in the range of 211–232, and the particles of the size (30 to infinity) and circularity value (0.2–0.8) of the objects were counted after excluding the edges. The numbers of the yeast cells and the hyphae were counted in the same way.

### Vitality

Vitality of *Candida* cells was determined by FungaLight™ Yeast CFDA-AM/Propidium Iodide Vitality Kit (Invitrogen). In this kit the acetoxymethyl ester (AM) moiety of the esterase substrate 5-carboxy-fluorescein diacetate (CFDA) aids the reagent to enter the cells, where in response to the intracellular esterase activity, it is converted to a product that emits a green fluorescent signal. On the other hand, propidium iodide (PI) can penetrate a dead cell trough the damaged membrane where it binds to DNA and produces a red fluorescent signal. Cells from 3 ml cultures were collected by centrifugation at 10000 g, 4 °C for 3 min, were resuspended in 300 μl PBS, were stained by adding 0.3 μl PI (20 mM stock solution) as well as 0.6 μl CFDA (0.1 mg/ml stock solution), and were incubated at 37 °C for 30 min with the reagents. After a quick vortexing 20 μl of the cell suspension were dropped on slides and were examined under a Leica SP8 confocal microscope. Intensity density was measured from the microscopic images of three independent biological replicates by ImageJ software. Five randomly selected fields of view were investigated for all samples. Image type was set to 8-bit grey scale, while threshold was adjusted to 13 for green and 26 for red channels. An automatic particle analysis was carried out after the elimination of the outliers.

### Flow Cytometry

Morphological analysis of *C. albicans* was confirmed by flow cytometry in Novocyte 3000 flow cytometer (Acea Biosciences, Inc.). Cells of three biological replicates were suspended in 300 μl of PBS and 500,000 events per sample were analyzed from each sample. The yeast and hyphae populations were identified on FSC-A versus FSC-H density plots with the doublet discrimination method [42].

### Antioxidant enzyme activities and glutathione concentration

Protein extracts were prepared from four independent biological samples as follows. *C. albicans* cells were collected by centrifugation and resuspended in 400 μl PBS. The cell suspensions were mixed with 500 μl slurry of *cc.* HCl treated, heat sterilized glass beads (1 mm Ø, Marienfeld) and were vortexed at the maximal speed (in ZX^3^ VELP apparatus) four times for 1 min with 2 min intervals of cooling in ice. Cells debris were removed by centrifugation at 10000 rpm, 4 °C for 8 min and were used immediately. Catalase, glutathione reductase, glutathione peroxidase, and superoxide dismutase activities of supernatants were assayed by photometric methods as described earlier by [43]. One unit of superoxide dismutase was defined as the amount of enzyme which inhibited the nitro blue tetrazolium oxidation reaction rate of the control (without enzyme) by 50% [22, 44]. The protein contents of cell-free extracts were determined by Bradford method [45]. For the estimation of glutathione contents, the fungal cells were washed with PBS, resuspended in 5% sulfosalicylic acid (Sigma), and stored at − 20 °C until use. Reduced (GSH) and oxidized (GSSG) glutathione concentrations were assayed by the glutathione reductase-DTNB [5, 5′-dithiobis-(2-nitrobenzoic acid)] rate assay of Anderson [46].

### RNA preparation and quality tests

Total RNA samples were prepared according to [40] with the exception that the lyophilization step was omitted in the case of the DNA chip hybridization. The quality of the RNA was tested by NanoDrop (Thermo Scientific) and either by agarose gel-electrophoresis (Bio-Rad) or by BioAnalyzer (Agilent) using Eukaryotic Total RNA Nano Kit according to manufacturer’s protocol. Only the samples with RNA integrity number (RIN) value > 7 were accepted for RNA-Seq. The efficiency of the oxidative stress treatments was estimated in pilot RT-qPCR experiments (see later). All of the RNA samples that passed the quality control criteria were stored at − 70 °C before use.

### DNA microarray hybridization

In a single pilot experiment, we tested by DNA chip hybridization if our experimental setup was suitable for the detection of significant changes in mRNA levels. For DNS chip studies, an Agilent 60-mer oligonucleotide high density array (GE 4x44K AMADID 066939 SurePrint microarray slide; Chromoscience Ltd., Gencsapati, Hungary) was constructed. Oligos were designed with the eArray software of Agilent (design number: CA5314L) based on the C_albicans_SC5314/ Assembly21 coding sequence dataset (www.candidagenome.org). Total RNA was isolated as described above from representative *t*BOOH treated and untreated cultures of the WT and KO strains. Cyanine-3 (Cy3) labeled cRNA was prepared according to Agilent’s One-Color Labeling protocol (version 6.7). Prenormalised microarray data were obtained with Agilent’s Feature Extraction software (version 12.0.3.1) using default one-color high density protocol. Prenormalised data were background corrected using the normexp+offset method [47] followed by quantile normalization between arrays [48] as in Smyth [49].

### RNA-sequencing

RNA-Seq libraries were prepared from total RNA using TruSeq RNA Sample preparation kit (Illumina) according to the manufacturer’s protocol. Briefly, poly-A RNAs were captured by oligo(dT) conjugated magnetic beads then the eluted mRNAs were fragmented at 94 °C. First strand cDNA was generated by random priming reverse transcription and after second strand synthesis step double stranded cDNA was generated. After repairing ends, A-tailing, and adapter ligation steps the adapter ligated fragments were amplified in enrichment PCR and finally sequencing libraries were generated. Sequencing runs were executed on Illumina NextSeq500 instrument using single read 75 bp sequencing for three independent sets of biological samples. Approximately 18–20 million reads were generated for each sample. Raw reads were aligned to the reference genome (C_albicans_SC5314/ Assembly22 genome version, downloaded from www.candidagenome.org). Tophat and Cufflinks bioinformatics tools were used for the mapping and generating bam files and RPKM values of A and B alleles. The RPKM values of the corresponding A and B alleles were merged and these merged values were used for downstream analysis using StrandNGS software (www.strand-ngs.com). Data were normalized by DESEQ1 algorithm, then hierarchical clustering and Principal Component Analysis (PCA) were performed for identifying outlier samples in the dataset. Cluster v3.0 (http://bonsai.hgc.jp/~mdehoon/software/cluster/software.htm#ctv) and Java TreeView v1.1.6r4 (http://jtreeview.sourceforge.net/) open source applications were used for generating heat maps.

### Reverse transcription and quantitative polymerase chain reaction

As a prerequisite of all transcript analysis the efficiency of the oxidative treatment was confirmed in pilot experiments by testing the expression of antioxidant enzyme coding genes like SOD1, CAT1 or SOD3 using RT-qPCR [40] with the primer pairs described in Additional file 2: Table S1. To find the suitable gene(s) of normalization three candidates (ACT1, HSP70, HPT1) were tested in the same way. The mRNA levels of ACT1 proved to be constant under all of our experimental conditions. The same result was obtained independently of the cDNA synthesis priming method, thus ACT1 was selected as a normalizing gene in all of our experiments. It has to be mentioned that reverse transcription for mRNA quantification was initiated by oligo(dT) primer (Promega) whereas for checking rRNA levels we used random primers (Promega). RT-qPCR primer pairs were designed with Primer3 v.0.4.0 software (http://bioinfo.ut.ee/primer3-0.4.0/) and were checked with Oligo Analyzer 1.0.2 for self-annealing and with Nucleotide BLAST (https://blast.ncbi.nlm.nih.gov/Blast.cgi) for specificity. All of the primer pairs anneal to sequences which are identical in the A and B alleles of the target gene/cDNA. The nucleotide sequences and calculated melting points of the oligonucleotide primers are summarized in Additional file 2: Tables S1–S2. All primer pairs were tested for efficiency and specificity by PCR on WT genomic DNA target. All of them produced a single band of expected size, thus were found to be appropriate for RT-qPCR experiments.

### Statistical analysis

#### Physiological and biochemical data processing

Growth values, colony numbers and diameters, viability and vitality test results, enzyme activities, as well as glutathione contents were presented as mean ± standard deviation, *n* = 3–5. Significant (*p* < 0.05) differences between mean values were tested by two-sided, two-sample Student’s *t*-test [50].

#### Evaluation of RNA-Seq data

To identify differentially expressed genes between conditions ANOVA test combined with Tukey post hoc test and Benjamini-Hochberg FDR for multiple testing corrections of the StrandNGS software package (www.strand-ngs.com) was used. Up- and downregulated genes were regarded as differentially expressed genes if the │log2 FC│ values were higher or smaller than one.

#### GO term analysis

Gene set enrichment analysis of up- and downregulated genes was carried out with the GO term finder tool (http://www.candidagenome.org/cgibin/GO/goTermFinder) using default settings for function, process, and component gene ontology (GO) terms. Only enrichments characterized by an adjusted *p* < 0.05 were considered to be significant (Additional file 3: Dataset S5).

#### Evaluation of RT-qPCR data

Gene expression changes in the tested samples relative to the reference samples were characterized with ΔΔCP (log_2_ relative transcription) values and were presented as mean ± standard deviation, *n* = 5. Significantly (p < 0.05) higher or smaller than zero relative expression values were determined by ANOVA test combined with Tukey post hoc test (www.strand-ngs.com).

#### Correlation analysis

Pairwise correlations between log_2_FC values obtained from DNA chip, RNA-Seq or RT-qPCR experiments were characterized with Pearson’s correlation coefficient [50].

## Supplementary information

**Additional file 1:Figure S1.** Effect of oxidative stress on the growth rate of wild type and phosphatase mutant *C. albicans*. **Figure S2.** Quality check of RNA-Seq data by cluster analysis. **Figure S3.** Quality control of RNA-Seq data by principal component analysis. **Figure S4.** Correlation between gene expression data obtained by RNA-Seq and DNA chip hybridization. **Figure S5.** Schematic representation of the steps of rRNA maturation in *Candida*. **Figure S6.** Effects of CaPPZ1 gene deletion and 1 h *t*BOOH treatment on rRNA maturation in *C. albicans.*

**Additional file 2:Table S1.** Oligonucleotide primers used for testing the expression of protein coding genes. **Table S2.** Oligonucleotide primers used for testing the expression and maturation of ribosomal RNA.

**Additional file 3:Dataset S1.** for Additional figures. **Dataset S2.** for Fig. 1. **Dataset S3.** for Table 1. **Dataset S4.** for Fig. 2. **Dataset S5.** for Table 2**. Dataset S6.** for Fig. 3. **Dataset S7.** for Table 3. DNA chip and RNA-Seq. **Dataset S8.** for Table 3. RT-qPCR.

## Data Availability

All of the raw DNA chip and RNA-Seq data sets are available in the Gene Expression Omnibus (GEO; http://www.ncbi.nlm.nih.gov/geo/) with the following accession number: GSE134060. The original experimental results supporting the figures and tables can be found in Additional file 3.

## Abbreviations

CaPPZ1: *Candida albicans* protein phosphatase Z1 gene
CaPpz1: *Candida albicans* protein phosphatase Z1 protein
CFDA: 5-carboxy-fluorescein diacetate
DNA chip: Deoxyribonucleic acid microarray
FSC-A: Forward scatter area
FSC-H: Forward scatter height
GPI: Glycerophosphoinositol
GSH: Reduced glutathione
GSSG: Oxidized glutathione
KO: *cappz1* knockout strain
KOt: *t*BOOH treated *ppz1* knockout strain
MSF: Major facilitator superfamily
PBS: Phosphate-buffered saline
PI: Propidium iodide
RNA-Seq: Ribonucleic acid sequencing
RT-qPCR: Real-time quantitative polymerase chain reaction
SOD: Superoxide dismutase
*t*BOOH: *tert*-butyl hydroperoxide
TF: Transcription factor
WT: Wild type strain
WTt: *t*BOOH treated wild type strain
YPD: Yeast Extract–Peptone–Dextrose medium
