# Supplementary material for: Deletion of the fungus specific protein phosphatase Z1 exaggerates the oxidative stress response in Candida albicans
Source: BMC Genomics. 2019 Nov 19;20:873. doi: 10.1186/s12864-019-6252-6 (PMC6862791; doi:10.1186/s12864-019-6252-6)
Supplement: Supplementary file 1 — Additional file 1:Figure S1. Effect of oxidative stress on the growth rate of wild type and phosphatase mutant C. albicans. Figure S2. Quality check of RNA-Seq data by cluster analysis. Figure S3. Quality control of RNA-Seq data by principal component analysis. Figure S4. Correlation between gene expression data obtained by RNA-Seq and DNA chip hybridization. Figure S5. Schematic representation of the steps of rRNA maturation in Candida. Figure S6. Effects of CaPPZ1 gene deletion and 1 h tBOOH treatment on rRNA maturation in C. albicans. [file 12864_2019_6252_MOESM1_ESM.pdf]

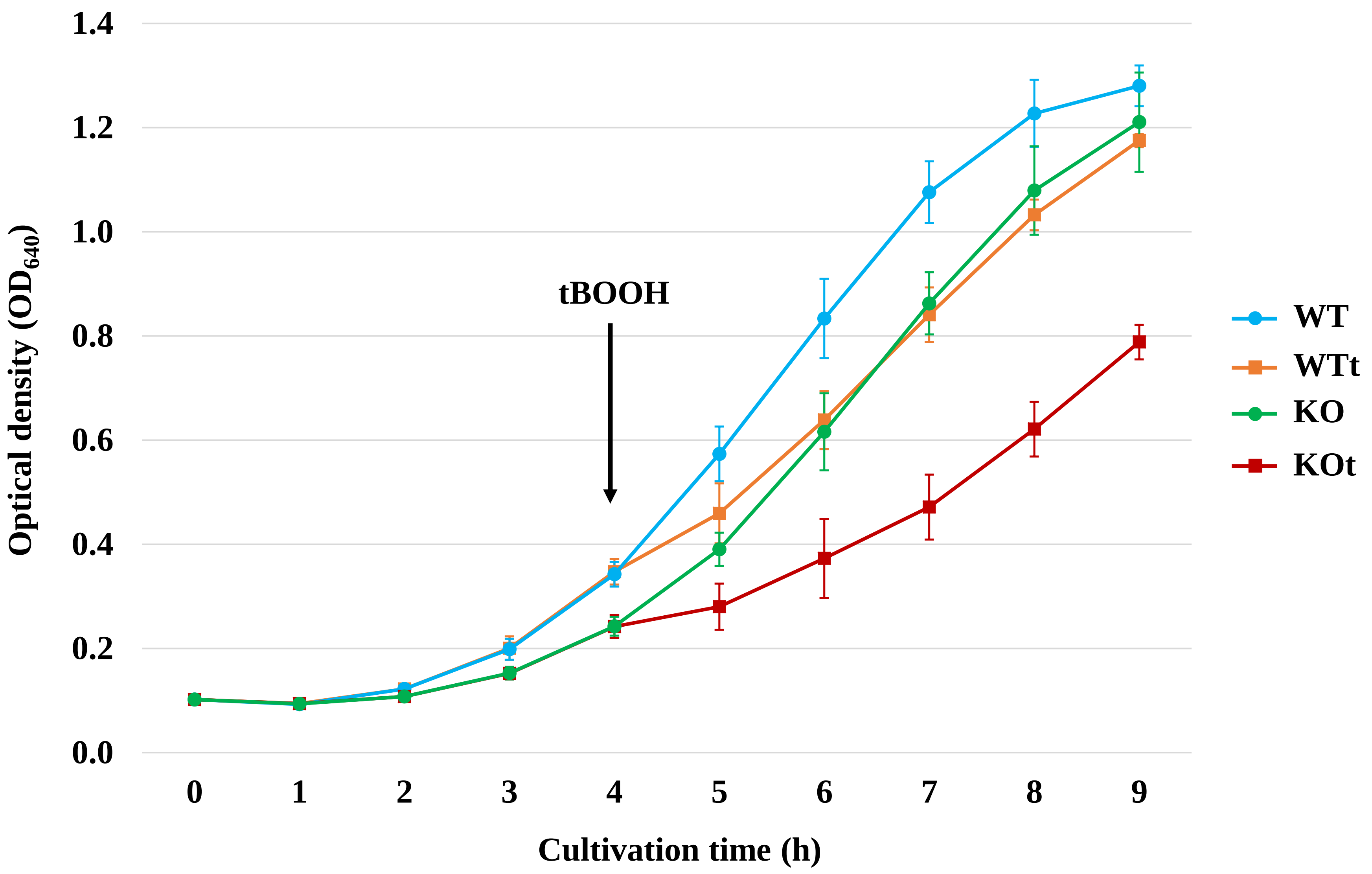

Figure S1. Effect of oxidative stress on the growth rate of wild type and phosphatase mutant *C. albicans*. The control QMY23 and the *cappz1* deletion mutant strains were compared either under normal growth conditions (WT and KO) or under oxidative stress induced by 0.4 mM tBOOH treatment (WTt and KOt). The growth rate of the samples was monitored by measuring their optical density at 640 nm before and after the addition of tBOOH at the fourth hour of cultivation. All of the data used in this figure can be found in additional file 3: Datasets S1.

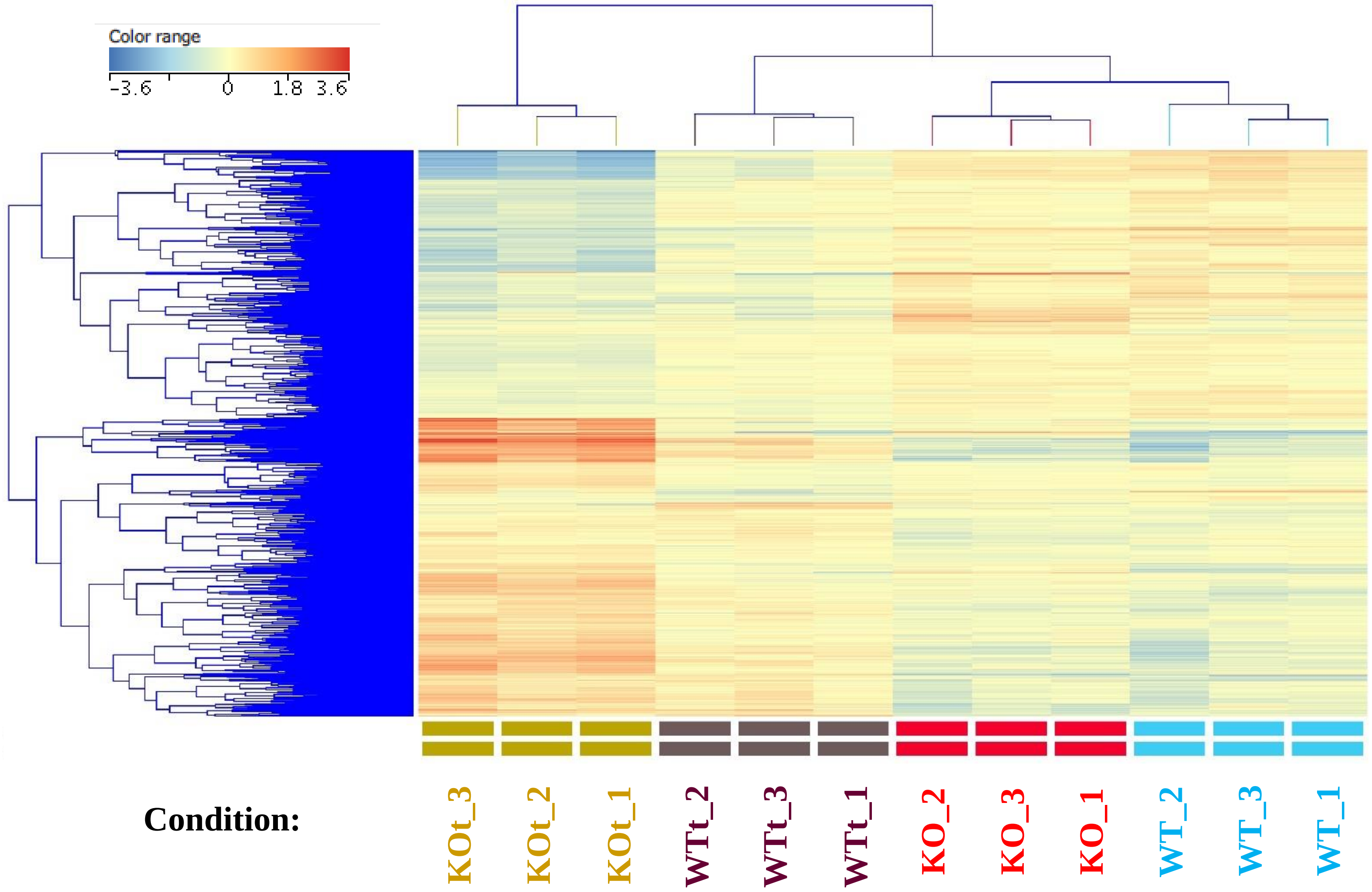

Figure S2. Quality check of RNA sequencing data by cluster analysis. The quality of RNA-Seq data was tested by clustering the gene expression patterns of 3 independent biological samples (1-3) under 4 different experimental conditions (KOt, WTt, KO and WT). The color scale represents the range of mean relative expressions in $\log_2$FC. The close clustering of the data obtained under identical conditions confirms the reproducibility of the experiments.

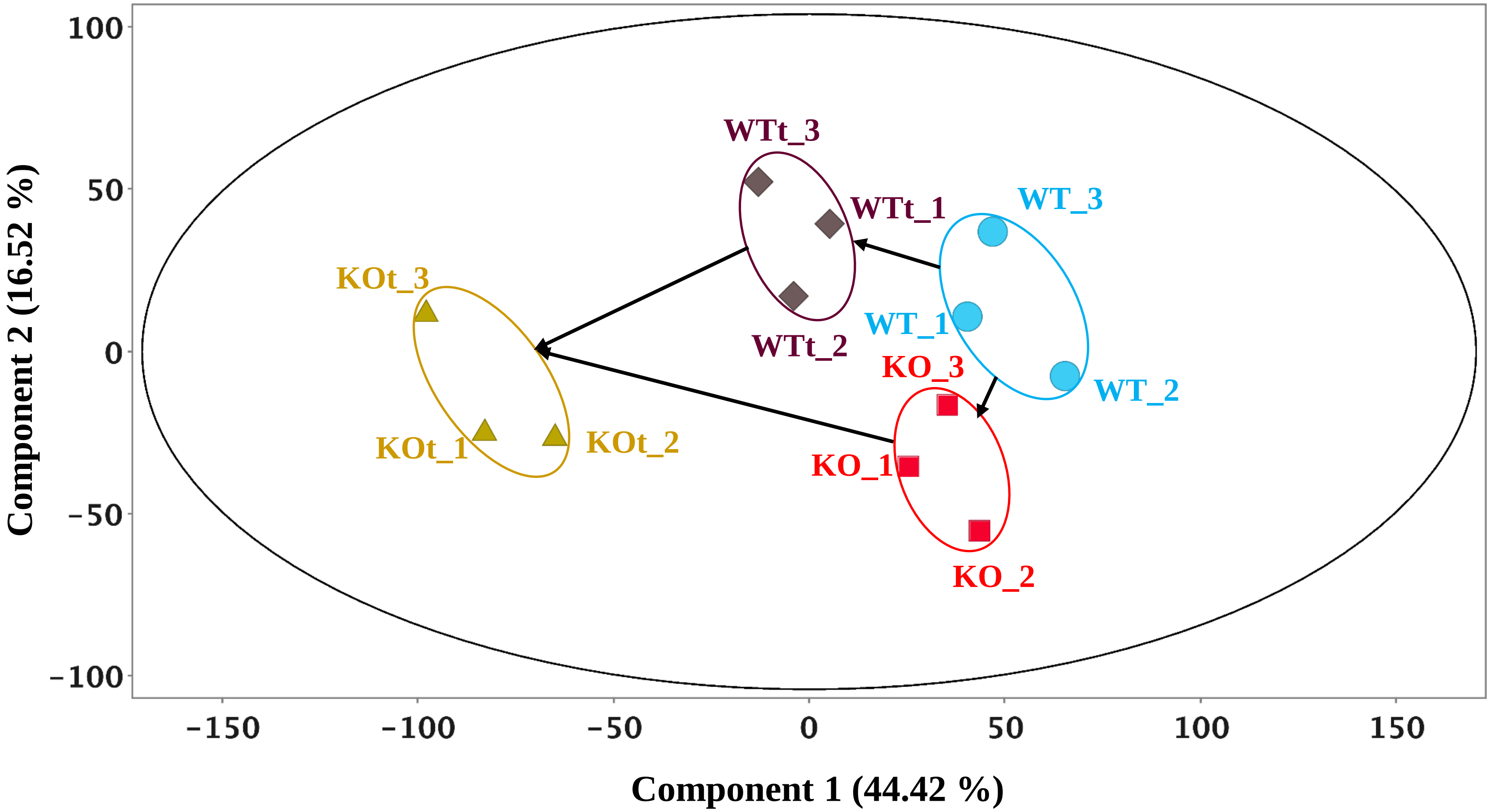

Figure S3. Quality control of RNA sequencing data by principal component analysis. The distribution of RNAseq data obtained with 3 parallel biological samples under 4 conditions described in Figure S4 was analyzed. The datasets of the three replicates of identical conditions (KOt, WTt, KO and WT) fall close to each other (circled). The contributions of the two main conditions are given in %. Arrows indicate the distances and the connections between the 4 conditions.

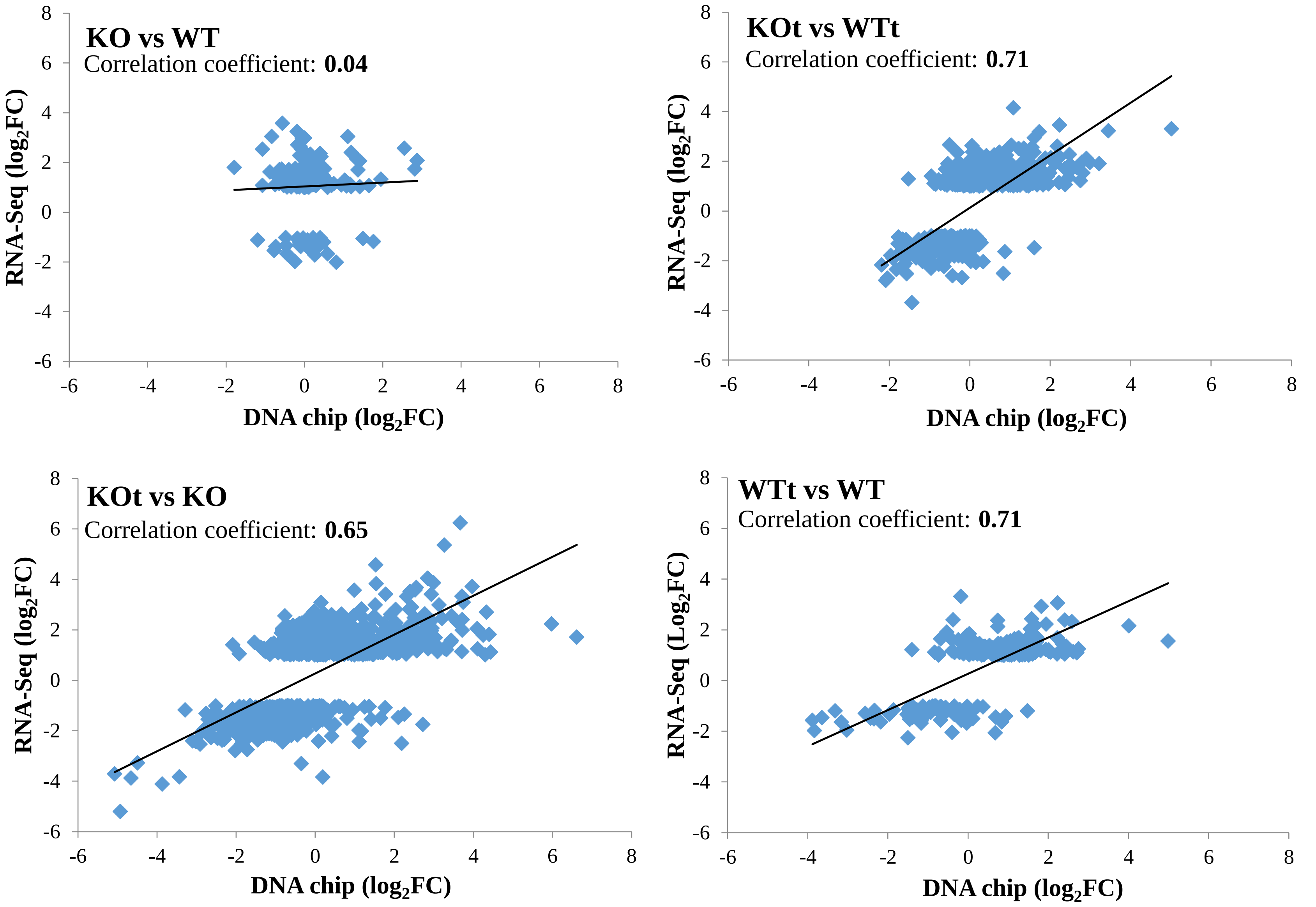

Figure S4. Correlation between gene expression data obtained by RNA-Seq and DNA chip hybridization. Average of relative gene expressions (in $\log_2$FC) obtained by two independent methods were compared in four relationships. The genes exhibiting less than two fold changes of expression in RNA-Seq experiments were excluded from the analysis. Pearson's correlation coefficients are included in each graph.

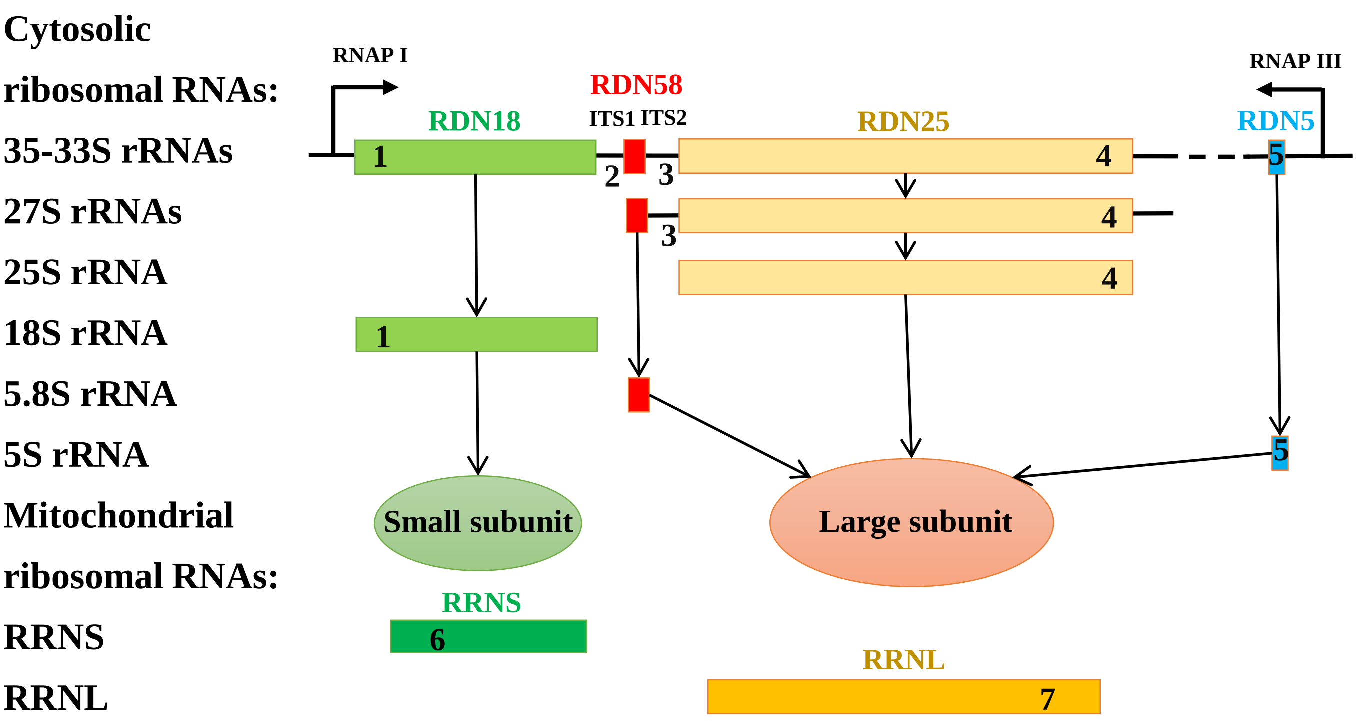

Figure S5. Schematic representation of the steps of rRNA maturation in *Candida albicans*. The 35S rRNA precursor is translated from the rDNA cluster by RNA polymerase I (RNAP I) and is processed in several subsequent steps to the 18S, 5.8S, and 25S rRNA species. The 5S rRNA is synthetized by RNA polymerase III (RNAP III) from another region of the cluster. The rRNA components of the mitochondrial ribosomes are transcribed from the mitochondrial genome by a specific polymerase. Numbers 1-7 label the binding sites of the primer-pairs (described in additional file 2: Table S2) that were used for the detection of specific rRNA segments.

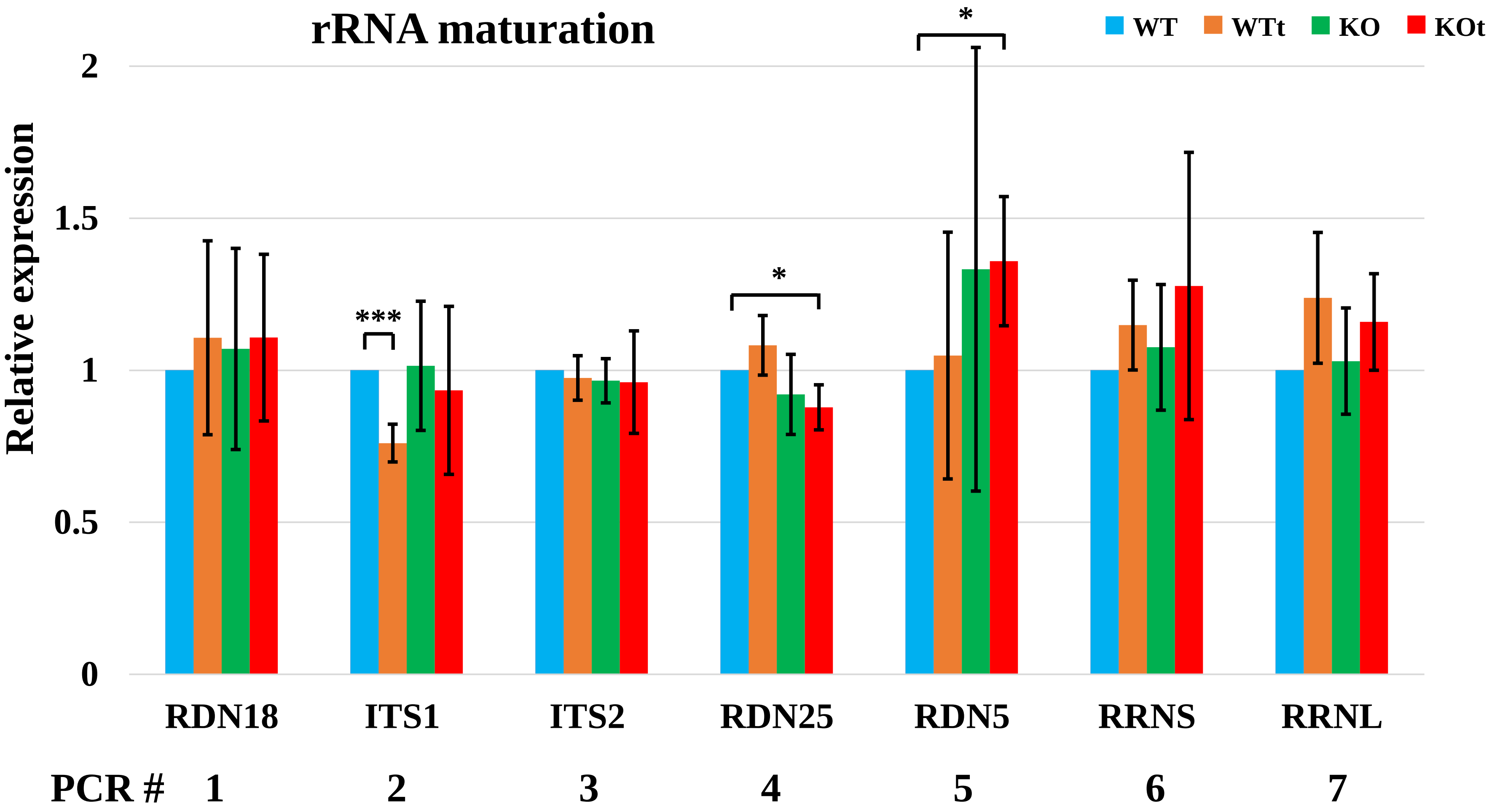

Figure S6. Effects of *CaPPZ1* gene deletion and 1h tBOOH treatment on rRNA maturation in. The relative expression of rRNA species was revealed by RT-qPCR with the PCR primer pairs # 1-7 that were introduced in Figure S5. The mean and SD of 5 independent biological replicates are shown. Values significantly different from one according to one sample Student's t-test are marked as * ($p < 0.05$), ** ($p < 0.01$), and *** ($p < 0.001$). All of the data used for the construction of this figure can be found in additional file 3: Datasets S1.
