# Supplementary material for: Deletion of the fungus specific protein phosphatase Z1 exaggerates the oxidative stress response in Candida albicans
Source: BMC Genomics. 2019 Nov 19;20:873. doi: 10.1186/s12864-019-6252-6 (PMC6862791; doi:10.1186/s12864-019-6252-6)
Supplement: Supplementary file 2 — Additional file 2:Table S1. Oligonucleotide primers used for testing the expression of protein coding genes. Table S2. Oligonucleotide primers used for testing the expression and maturation of ribosomal RNA. [file 12864_2019_6252_MOESM2_ESM.pdf]

Table S1. Oligonucleotide primers used for testing the expression of protein coding genes*

Gene name	Gene ID	Upper (U) primer (5'-3')	Lower (L) primer (5'-3')	Tm U - L (C°)**	Product (bp)
ACT1	C1_13700W	GTTGCTCCAGAAGAACATCC	ACCATCACCAGAATCCAAAAC	61.3-61.4	192
AOX2	C1_09150W	TGCCAATCCAAACACTTCAG	TGAGGAAACACTGGATGTGG	63.7-63.7	123
BUD22	CR_00680W	ACCAGAGGATGCCCAAGTAG	TATCGTCCACCGGGATAGTC	63.0-63.6	122
C3_02750W	C3_02750W	TTGATGTCCCCGTTAGAAATG	TTACTGGCAATGGTAGCATTC	63.5-61.5	127
CAM1	C3_06010W	TGTCTTTATTTGATTAACTTGGCTG	AACTCTAGCTTCAGTTGGCAAC	62.0-61.4	129
CAT1	C1_06810W	TTTCTCTTTTGGGTGTGGATG	GGTGGTGAATTAGGTTCTGC	63.7-61.0	164
CDC19	C2_05460W	TCCACCAAACCACGAAATG	ACCTGGAGCAATGACTTTGG	64.5-64.0	112
CDR1	C3_05220W	AAGATGTCGTCGCAAGATG	TCACTTGTATGGGCATCAAAC	61.5-62.4	110
CFL2	C4_05780C	GGTGTTGTCGCTACCATTTG	CACCATACCCTTGGTCGTC	63.0-62.9	157
CFL4	C5_01360W	AAACTATCCCCATTGATGTGC	CAATTGACAATAGCCCCAATAC	62.5-61.9	166
CHT3	CR_10110W	CCTCCACAGCACCAACTTC	TAACAACTGGGGTTGTCTCG	63.5-62.6	111
DIM1	C1_13730C	CCGGGTTCAAATCTTCTAATG	AAACCCAGTTTCAGTCAAAACAG	62.1-63.1	170
EFT2	C2_03100W	ACAAGATGGTTGCTTTCACTATTG	TTGGCAGCGGAAATAATACC	63.0-63.5	154
ENA2	C1_00390W	GTGCTGGAAATGGTGACTTG	CCAAGTCATTGAGGCAAATG	63.2-63.1	110
ENA21	C7_02910W	TGGCAGAAAATGTCGCTC	TATCGTCATCGGCACCAC	62.7-63.4	168
ENP1	C7_03700C	ATCCCAAGGTGGGAATTTAAG	ACCGCCACCATTTTCTTC	62.7-62.5	121
FET31	C6_00480C	TCTCATGTATTGCCGGTGTC	AAGAAGAAGATCCTTCATTGACG	63.6-62.4	150
FGR41	C1_10400C	AGAAAGTCCCGTGGAACAAG	ACTGGGGTTGGGGTAGAAG	63.0-62.7	114
GAP1	C5_02790C	GGGGATCATATTTTGGGTTAAC	CCCAAATATGCCAAAAAGAAATC	61.9-64.0	118
GAP2	C3_05580C	TTGTACAGTTATGTCCCTTGGG	CACCACTAGCCATTGCATAGC	62.5-64.4	139
GCV2	C1_08400C	TCTTGAATCCCCTGAGTGG	TCACCAGCAGCAGTACCTTC	62.7-63.1	163
GIT1	C2_06590C	ATGTGTGCTGCTTCTCATGG	TTAGCAGCTTCACTTGCTGTC	64.0-62.3	131
GLR1	C5_01520C	TTTTCCGCTGACAAGACTTTG	ATTCAACCCCAATGTAACCAG	64.1-62.3	160

Table 1. continued					
**Gene name**	**Gene ID**	**Upper (U) primer (5’-3’)**	**Lower (L) primer (5’-3’)**	**Tm U - L (C⁰)****	**Product (bp)**
GPM1	C2_03270W	ATCCAGAGCCATCCAAACTG	CTTGACCGTAAGCTTCCAAAG	64.0-62.6	149
HGT1	C1_01980W	GTGTCACCGTTCCTGCATTATAT	AAGGAATTGAGTATTGACCCAAA	63.6-62.3	129
HGT12	C7_00280W	TTGTTGGGCTATTTGTGGTG	CACCTTTGAACCCAAGTCG	63.3-63.2	160
HPT1	C2_02740C	TGAAAAGCCAGGTACCCAAG	TGCGTAATGCAAAGTGGTTC	63.9-63.6	130
HSP70	C1_1348W	CATTAGTGGTGCTTATGGTGC	ACTTCTTCAACAGTTGGTCCA	61.9-61.2	164
IFF11	C3_00600W	AATCTCTGTCGCCATTTTGG	TCCAAGGCAATACACCCAC	63.9-63.4	123
IFM1	C7_02940C	ATACACCTGGACACGCTGC	CGATGGTTTGAGGCATAACC	63.9-64.1	111
JEN2	C4_04030W	TACTTCTGGGCATTCCATCC	GAACGGAAATGTGGAGGAG	63.7-62.1	112
MET13	C3_02950C	ACGTAAAGGGAGCCGAATC	TTCTCAACGATTGTGGGTTG	62.9-63.6	157
MRPL3	C1_07910C	GGGAGATTATCGGCTCATCC	TTTATTGGTTCACCATTTGCAC	64.5-63.3	170
NAG3	C6_04610C	ACACCATTCACCATGACGAC	GTTCCAATGGCCCTAACTG	63.5-61.9	117
NSA2	C3_06380W	AAGGGTTGGAGAGCCAAAC	ATACGTTGGCAAGGCCTC	63.0-62.7	144
OYE23	C6_01510W	TTGGGATTTTTGATGCTTCG	TGCAAACCCCACAATAGTACG	64.7-64.5	168
PGK1	C6_00750C	AACGATGCCTTTGGTACTGC	GGGTTTTCCAAAGCCTTAGC	63.9-63.6	125
PHO84	C1_11480W	GTTTGTTGGGTTTGTTCGTC	ATGGCACCGACTTTACCAG	61.8-62.6	148
PLB1	C6_01990W	AAAATTAGACAATGGTCGTCCA	AGGTATGTTAGCTTCAGTGATAGACAAC	62.1-62.6	127
PPZ1	CR_06420W	TCACAACAACGTCCACAACCA	GACAACGGTGAACCTGGC	64.7-64.4	173
RPL29	C1_11040W	CAGCTCACAACCAAACCAG	AAAGCTTTGGCAGTACCATG	62.0-61.7	139
RPL5	C7_01790C	ATTTTCGGTGGTCACGTTG	GGTCAGCTCTAATGGCTTCG	63.4-63.8	157
RPP1B	C7_03920C	CTCCGTCTCATACGCTGC	TTTACCTTCAACGGCTTTGG	62.3-63.7	142
RPS3	CR_04810W	CAAGGTTATGCTGGTGTTGAAG	AAATGGCAATACCTTCTGGAG	63.3-61.8	166
RPS8A	C2_05610C	CACAAAAGATCCGCCACTG	CCGGTTTCAACTCTCAAAGC	64.3-63.6	164
RRN3	C2_00030W	CAGCGAAATCTTGGAAAAGC	GTTGAGCCAACCGACAAG	63.6-62.0	124
RSM26	C5_04530W	CTTGGCATGGACCGATTATC	TAGTTTGCTTGGCTGTGTGC	64.1-64.0	119
SOD1	C4_02320C	TTGAACAAGAATCCGAATCCG	GAAATGAGGACCAGCAGAAG	65.9-61.4	137

Table 1. continued					
**Gene name**	**Gene ID**	**Upper (U) primer (5’-3’)**	**Lower (L) primer (5’-3’)**	**Tm U - L (C⁰)****	**Product (bp)**
SOD3	C7_00110W	TGCTCAGTATGGGTCTGTTTC	ATCCTGATTGGCAGTAGTGAC	61.7-60.6	142
SOD4	C2_00660C	GGATTGCCTTCAGGTGTTG	AGCTGGTGTTTTGGCAGTAG	63.2-61.9	133
SPB4	C5_01600C	ATCTCTTGCATGGGAGAATTTAC	CCTGATCCAGTGACTGCTTC	62.1-62.5	159
TRK1	CR_07960C	TGAAGAGCCACAGAGACCAAG	TCAGGGTCAATCGACTTCC	64.5-62.8	109

*All of the primers listed here hybridize equally to the A and B alleles of the target cDNA.

**Data provided by the manufacturer (Sigma).

Table S2. Oligonucleotide primers used for testing the expression and maturation of ribosomal RNA

#	Gene name	Gene ID	Upper (U) primer (5'-3')	Lower (L) primer (5'-3')	Tm U - L (Cº)*	Product (bp)
**1**	RDN18	CR_08770W	CGACTGTTTGGAAGGGATG	TGAAACCATGGTAGGCCAC	63.2-63.4	167
**2**	ITS1	CR_08780W	CGGAAGGATCATTACTGATTTG	CAAGAGATCCGTTGTTGAAAGT	62.1-62.0	177
**3**	ITS2	CR_08800W	CATGCCTGTTTGAGCGTC	CCTGATTTGAGGTCAAAGTTTG	63.2-62.4	191
**4**	RDN25	CR_08810W	TCGGTAAGCGTTGGATTG	AGCCGCAAAAACCAATTATC	62.3-62.6	160
**5**	RDN5	CR_08760C	TGCGGCCATATCTAGCAG	GCACAATAGTTTCGCGTATGG	62.6-64.1	110
**6**	RRNS	CM_00360W	CCAGCAGTAGCGGTCATACAC	TCTGGAGGACGGGCTATC	64.5-62.9	120
**7**	RRNL	CM_00010W	TGGTCGTCTAGGCTCAACC	GAGCCACATGCAACAGATG	62.9-63.1	134

*Data provided by the manufacturer (Sigma).
